# Small RNAs in parasitic nematodes – forms and functions

**DOI:** 10.1017/S0031182019001689

**Published:** 2020-07

**Authors:** Collette Britton, Roz Laing, Eileen Devaney

**Affiliations:** Institute of Biodiversity, Animal Health and Comparative Medicine, College of Medical, Veterinary and Life Sciences, University of Glasgow, Bearsden Road, Glasgow G61 1QH, UK

**Keywords:** Extracellular vesicle, gene regulation, microRNA, nematode, parasite, Piwi-interacting RNA, small interfering RNA

## Abstract

Small RNAs are important regulators of gene expression. They were first identified in *Caenorhabditis elegans*, but it is now apparent that the main small RNA silencing pathways are functionally conserved across diverse organisms. Availability of genome data for an increasing number of parasitic nematodes has enabled bioinformatic identification of small RNA sequences. Expression of these in different lifecycle stages is revealed by small RNA sequencing and microarray analysis. In this review we describe what is known of the three main small RNA classes in parasitic nematodes – microRNAs (miRNAs), Piwi-interacting RNAs (piRNAs) and small interfering RNAs (siRNAs) – and their proposed functions. miRNAs regulate development in *C. elegans* and the temporal expression of parasitic nematode miRNAs suggest modulation of target gene levels as parasites develop within the host. miRNAs are also present in extracellular vesicles released by nematodes *in vitro*, and in plasma from infected hosts, suggesting potential regulation of host gene expression. Roles of piRNAs and siRNAs in suppressing target genes, including transposable elements, are also reviewed. Recent successes in RNAi-mediated gene silencing, and application of small RNA inhibitors and mimics will continue to advance understanding of small RNA functions within the parasite and at the host–parasite interface.

## Introduction

**Fig. 1.**
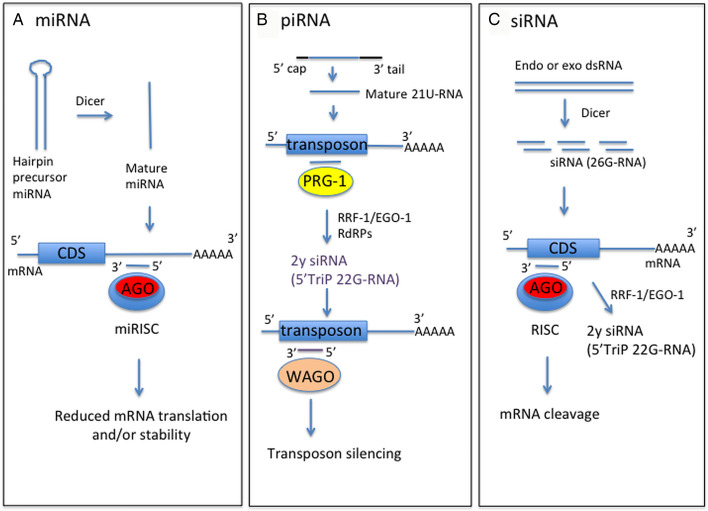
Schematic of forms and functions of small RNA classes in nematodes, based on *C. elegans* information. (A) Mature miRNA strand, derived from precursor miRNA, is incorporated into the miRNA-induced silencing complex (miRISC) containing Argonaute protein (Ago). This complex directs binding to mRNA target sequences, commonly in the 3′-UTR. Binding specificity is determined by complementarity between the target sequence and miRNA seed sequence (nucleotides 2–7). (B) Mature piRNA (21U-RNA) are processed from a capped precursor and bind to Piwi Argonaute PRG-1 to recognize target sequences, often transposons, by imperfect complementary base-pairing. This initiates synthesis of secondary small inhibitory RNAs (siRNAs) with 5′ triphosphate (5′TriP 22G-RNAs) by RNA-dependent RNA polymerases (RdRPs) RRF-1 or EGO-1. 22G-RNAs associate with worm-specific Argonaute proteins (WAGOs) to mediate target silencing. (C) Endogenous or exogenous dsRNA is processed by dicer into siRNAs, which bind anti-sense to mRNA exonic sequence to mediate mRNA cleavage by RDE-1 Argonaute. siRNAs also act as primers for synthesis of 22G-RNAs by RRF-1 or EGO-1 to amplify the RNAi response, using target dsRNA as a template.

Knowledge of small RNA structure and function has increased greatly in the last decade. The free-living nematode *Caenorhabditis elegans* led the way, with the initial discovery of microRNAs (miRNAs) and small interfering RNAs (siRNAs) in this species (Lee *et al*., 1993; Reinhart *et al*., 2000; Fire *et al*., 1998). The subsequent identification of miRNAs in diverse organisms using experimental and/or bioinformatics approaches (Pasquinelli *et al*., 2000; Lagos-Quintana *et al*., 2001; Lau *et al*., 2001; Lee and Ambros, 2001), and the discovery of siRNA gene silencing pathways in plants (Hamilton and Baulcombe, 1999), established the important roles that small RNAs play in suppressing gene expression. This review focuses on small RNAs in nematodes and what we know about these from genome, transcriptome and functional studies. Much progress has been made recently in characterizing nematode miRNAs and these are discussed in most detail. miRNAs were initially identified as regulators of nematode development (see below), however their presence in parasite excretory-secretory (ES) products has stimulated interest in these molecules as modulators of host–parasite interactions to promote parasite survival. The ability of miRNAs to alter levels of gene expression suggests they could have multiple, as yet undefined roles, in parasitic nematode biology. For example, altered expression of miRNAs may be associated with anthelmintic resistance (Devaney *et al*., 2010; Gillan *et al*., 2017), akin to changes in miRNA levels observed in drug-resistant tumour cells (reviewed in Ghasabi *et al*., 2019). This review will also discuss nematode Piwi-interacting RNAs (piRNA), required for silencing of transposable elements in the germline, but of which little is currently known in parasitic species. We also discuss the forms and functions of siRNAs, recent successes in RNA interference (RNAi)-mediated gene silencing in parasitic nematodes, and the application of this in progressing from genome to function. A diagram summarizing different small RNA classes discussed in this review is presented in Fig. 1.

## MicroRNAs

### miRNA discovery

miRNAs regulate gene expression post-transcriptionally by binding with partial sequence complementary to the 3′-untranslated region (UTR) of their target mRNAs (Bartel, 2009, 2018). This interaction inhibits protein translation and results in miRNA degradation (Chekulaeva and Filipowicz, 2009). The first miRNAs, *lin-4* and *let-7*, were discovered in *C. elegans* (Lee *et al*., 1993; Reinhart *et al*., 2000). The subsequent availability of genome data for a range of vertebrates and invertebrates revealed conservation of miRNA-mediated gene regulation (Pasquinelli *et al*., 2000; Lagos-Quintana *et al*., 2001; Lau *et al*., 2001; Lee and Ambros, 2001). Their conservation in diverse organisms has benefited parasitic nematode miRNA research through development of bioinformatics databases, such as miRBase (Release 22.1; http://www.mirbase.org/) (Griffiths-Jones *et al*., 2008), miRCarta (Version 1.1; https://mircarta.cs.uni-saarland.de/) (Backes *et al*., 2018), miRGeneDB (Version 2.0; http://mirgenedb.org/) (Fromm *et al*., 2015, 2020) and target prediction programmes [e.g. TargetScan (Lewis *et al*., 2005)], together with advancements in miRNA mimic and inhibitor chemistry. For example, mammalian *mir-122*, expressed in hepatocytes, is required for hepatitis C virus accumulation and a locked nucleic acid-modified oligonucleotide complementary to *mir-122* can suppress viral load and is being evaluated as a therapeutic treatment (Titze-de-Almeida *et al*., 2017). In addition, specific miRNAs released into plasma or urine are being studied as potential diagnostic/prognostic biomarkers of diseases, including cancer (Wang *et al*., 2018), and filarial nematode infections (Buck *et al*., 2014; Tritten *et al*., 2014*a*, 2014*b*; Quintana *et al*., 2015).

miRNAs are derived from long primary transcripts that are processed to precursor miRNAs of approximately 70 nucleotides that fold into a hairpin structure (Kim *et al*., 2009; see Fig. 1). Mature miRNAs are 20–26 nucleotides in length (Fromm *et al*., 2015). Nucleotides 2–7 are referred to as the seed sequence and are important in determining the specificity of binding to target mRNAs. Novel miRNAs are most often identified by sequencing of small RNA libraries, mapping the sequences to the genome, where available, and testing that the region each miRNA is derived from folds into a hairpin structure, using programmes such as RNAfold (Hofacker *et al*., 1994). Discovery pipelines, such as miRDeep2 (Friedländer *et al*., 2012), incorporate RNAfold and other scoring criteria, and have been applied to miRNA discovery and validation in a range of parasitic nematodes, as summarized in Table 1.

**Table 1.** Animal and human parasitic nematode species for which miRNA data are available from parasite extracts, EVs, ES supernatant or released into host serum/plasma. From small RNA sequencing data, unless indicated otherwise

Nematode species (clade)	Developmental stage/host sample	Small RNA class	GEO Acc. No./in MiRBase	Reference
*Angiostrongylus cantonensis* (V)	Male and female adults	miRNA, siRNA	NA/No	Chen *et al*. (2011*a*)
*Ascaris lumbricoides* (III)	Female adults	miRNA	NA/No	Shao *et al*. (2014)
*Ascaris suum* (III)	Germline, zygote, embryo, L1–L3	miRNA, siRNA	GSE 26956, GSE 26957/Yes	Wang *et al*. (2011)
	Male and female adults	miRNA	NA/No	Xu *et al*. (2013)
	Female adults	miRNA	NA/No	Shao *et al*. (2014)
*Brugia malayi* (III)	Male and female adults, mf	miRNA, siRNA	NA/Yes	Poole *et al*. (2010)
	Male and female adults, mf	miRNA	NA/Yes	Poole *et al*. (2014)
	L3 EV	miRNA	SRA PRJNA 285132/No	Zamanian *et al*. (2015)
*Brugia pahangi* (III)	L3, mixed sex adults	miRNA, siRNA	GSE 34539/Yes	Winter *et al*. (2012)
	L3, L4, male and female adults	miRNA microarray	NA/No	Winter *et al*. (2015)
	Infected dog plasma	miRNA	NA/No	Tritten *et al*. (2014*a*)
*Dirofilaria immitis* (III)	Mixed sex adult worms	miRNA	GSE 35646/No	Fu *et al*. (2013)
	Infected dog plasma	miRNA	NA/No	Tritten *et al*. (2014*a*)
*Haemonchus contortus* (V)	L3, mixed sex adults	miRNA, siRNA, piRNA	GSE 34539/Yes	Winter *et al*. (2012)
	L3, L4, male and female adults, gut	miRNA microarray	GSE 101501/No	Marks *et al*. (2019)
	L4 EV and ES, adult EV and ES	miRNA	NA/No	Gu *et al*. (2017)
*Heligmosomoides polygyrus* (V)	Egg, L3, adults, adult EV and ES	miRNA, siRNA, piRNA, YRNA	GSE 55941/Yes	Buck *et al*. (2014)
*Litomosoides sigmodontis* (III)	Infected mouse serum	miRNA	GSE 55978/No	Buck *et al*. (2014)
*Loa loa* (III)	Infected baboon plasma	miRNA	NA/No	Tritten *et al*. (2014*b*)
*Onchocerca ochengi* (III)	Infected cow plasma	miRNA	NA/No	Tritten *et al*. (2014*b*)
	Infected cow nodule fluid	miRNA	GSE 63933/No	Quintana *et al*. (2015)
*Onchocerca volvulus* (III)	Infected human serum	miRNA	NA/No	Tritten *et al*. (2014*a*)
	Infected human serum	miRNA	GSE 63933/No	Quintana *et al*. (2015)
*Strongyloides ratti* (IV)	Infective L3 and mixed stage	miRNA	GSE 41402/Yes	Ahmed *et al*. (2013)
*Toxocara canis* (III)	Male and female adults	miRNA	GSE 68710/No	Ma *et al*. (2016)
*Trichinella spiralis* (I)	Muscle stage larvae	miRNA	NA/No	Chen *et al*. (2011*b*)
*Trichuris muris* (I)	Mixed sex adult EV	miRNA	GSE 93667/No	Tritten *et al*. (2017)

We applied a small RNA library sequencing approach to identify small RNAs from the clade V ovine gastrointestinal nematode (GIN) *Haemonchus contortus* and the clade III filarial parasite *Brugia pahangi* (Winter *et al*., 2012). Our data showed that while some miRNAs were conserved across diverse organisms or throughout the nematodes, many were unique to these species. As genome annotation improves for parasitic nematodes, it is likely that some of these unique miRNAs may be found to be clade or niche specific. However, it does suggest that miRNAs are evolving rapidly, perhaps reflecting roles in host–parasite interactions. In general, *H. contortus* novel miRNAs were found to be expressed at a low level compared to conserved miRNAs, based on small RNA sequencing (Winter *et al*., 2012) and microarray data (Marks *et al*., 2019). This is consistent with findings in other organisms (Liang and Li, 2009; Shen *et al*., 2011) and suggests there is conservation of the machinery regulating miRNA gene expression, of which little is currently known. More recent work has identified motifs within the upstream promoter region of *C. elegans* miRNA genes that may determine expression level (Jovelin *et al*., 2016).

### miRNAs in parasitic nematode development

In *C. elegans*, miRNAs *lin-4* and *let-7* were discovered through genetic studies, based on their essential roles in regulating genes involved in development. *lin-4* suppresses expression of heterochronic gene *lin-14* to allow progression from larval stage L1 to L2 (Lee *et al*., 1993), while *let-7* modulates gene expression to promote adult development (Reinhart *et al*., 2000). For parasitic species, sequencing or microarray analysis can identify miRNAs expressed in different lifecycle stages, to help determine their roles in regulating development. However, we currently know little of the functions of parasite miRNAs and the genes they modulate. As mentioned above, bioinformatics programmes are available to predict interactions between specific miRNA sequences and target mRNAs. Some of these employ experimental validation, such as mirWIP, which incorporates immunoprecipitation (IP) data, using antibodies to miRISC (RNA-induced silencing complex) proteins AIN-1 and AIN-2, to score miRNA target sites (reviewed in Ambros and Ruvkun, 2018). However, currently many programmes rely on custom databases designed for specific species, such human, mouse, *C. elegans*, zebrafish (e.g. TargetScan; http://www.targetscan.org/vert_72/), with only a few programmes available that allow input of a test miRNA and 3′-UTR sequence. In addition, current assembly and annotation of most parasitic nematode genomes is not of sufficient quality to allow reliable identification of 3′-UTR sequences to predict miRNA binding sites. We were able to generate 3′-UTR datasets for *H. contortus* (Gillan *et al*., 2017), due to the advanced nature of the genome data (Laing *et al*., 2013). With developments in technology, such PacBio long read sequencing of RNA libraries (Iso-seq), improved annotation of UTRs in genomes of other nematode species should be feasible.

By investigating stage-specific miRNAs using microarrays, we identified the potential roles of some miRNAs in *H. contortus* and *B. pahangi* development. Enrichment of specific miRNAs was found in pre- and post-infective larval stages and in adult male and female worms for both species (Winter *et al*., 2015; Marks *et al*., 2019). For *H. contortus*, we focused on two miRNAs that were significantly enriched in the arrested L3 stage. Target prediction and gene ontology analysis suggested that these miRNAs suppress metabolism to maintain an arrested state. Comparative functional studies using *C. elegans* mutants indicated that the two miRNAs may suppress development by synergizing with DAF-16 FOXO transcription factor (TF) activity (Marks *et al*., 2019). By combining these different approaches, we progressed from initial identification of miRNAs for determining their expression patterns, and predicting target genes and potential roles in regulating development. miRNAs often act to fine-tune gene expression and can regulate key switches in developmental pathways. Therefore, identifying the potential target genes and pathways regulated by miRNAs is important in improving understanding of parasitic nematode development and could prove useful in designing novel therapeutic interventions.

Microarray analysis was similarly informative in identifying enrichment of miRNAs in specific lifecycle stages of the filarial nematode *B. pahangi*, from mosquito-derived L3, through to L4 and male and female adult worms (Winter *et al*., 2015). We focused on *B. pahangi mir-5364*, which is significantly upregulated in the post-infective L3 within 24 h of infection of a mammalian host and is a novel member of the *let-7* family. Target prediction programmes identified transcripts encoding several putative TFs, and interaction between *bpa-mir-5364* and the 3′-UTR of these mRNAs were confirmed experimentally using dual luciferase reporter assays. Further analysis of differentially expressed *B. pahangi* and *H. contortus* miRNAs and their target genes will help reveal the roles that miRNAs play in regulating nematode development at key points in infection.

### miRNAs in host–parasite interactions

In recent years there has been an explosion of interest in extracellular vesicles (EVs). These are small vesicles, between 50 and 200 nm in size, released by cells and are considered to be important for intercellular communication. EVs are released by a range of cell types, including tumours, and their uptake by recipient cells may alter cellular activity. Advances in protein and RNA sequencing technology, combined with genome data, have allowed detailed analysis of EV cargo from mammalian cells and from parasitic nematodes (Buck *et al*., 2014; Zamanian *et al*., 2015; Tzelos *et al*., 2016; Gu *et al*., 2017; Tritten *et al*., 2017, Hansen *et al*., 2019). miRNAs and to a lesser extent Y RNAs (small non-coding RNAs involved in RNA quality control and DNA replication), have been identified within parasitic nematode EVs (Buck *et al*., 2014). It is speculated that packaging of small RNAs within EV may protect them from degradation and facilitate their uptake by other cell types.

Buck *et al*. (2014) first identified miRNAs in the ES supernatant and within EV released by adult worms of the mouse GIN *Heligmosomoides polygyrus* during *in vitro* culture. Notably, within EV there was enrichment for miRNAs with identical seed sequences to mammalian miRNAs, including *mir-100*, *let-7*, *lin-4* and *bantam*. Subsequent small RNA sequencing of ES supernatant and EV released *in vitro* by *H. contortus* L4 and adult worms (Gu *et al*., 2017), *B. pahangi* L3 (Zamanian *et al*., 2015) and *Ascaris suum* adults (Hansen *et al*., 2019) also found enrichment of some of the same miRNAs. This raises the interesting possibility that parasite miRNAs may be mimicking or hijacking host cell gene regulation, perhaps for their own benefit (Buck *et al*., 2014). Notably, the helminth-associated cytokine IL-13 was identified as a predicted target of *A. suum lin-4* and *let-7* (Hansen *et al*., 2019). From an evolutionary perspective, as the sequences of these abundant, secreted miRNAs are conserved between the parasite and the host, avoidance of this putative parasite manipulation of host genes through mutation is less likely to occur (Claycomb *et al*., 2017).

While presence within EV could reflect higher abundance of specific miRNAs, there does seem to be selectivity in what is loaded into EVs. Some miRNAs, abundant in somatic tissue, are not present in EVs and studies in mammalian cell systems also suggest that the EV profile is not a snapshot of total cellular miRNAs (Driedonks *et al*., 2018). Determining how selectivity is achieved and the mechanisms by which parasite miRNAs are loaded into EVs require further work. This is likely to be guided by data from mammalian cell culture systems, demonstrating that RNA binding proteins recognize specific motifs that dictate miRNA exosomal sorting (Villarroya-Beltri *et al*., 2013; Shurtleff *et al*., 2016). EV released by adult worms of both *H. polygyrus* and *H. contortus* show enrichment of miRNAs homologous to miRNAs expressed in *C. elegans* gut cells (e.g. *mir-60* and *mir-236*; Martinez *et al*., 2008) and also identified in the gut of *H. contortus* (Marks *et al*., 2019) and of the pig GIN *A. suum* (Gao *et al*., 2017). This suggests that, at least for some nematodes, EV miRNAs may be derived from the gut, perhaps reflecting the metabolic activity of this tissue (Buck *et al*., 2014). In contrast, immunolocalization using an antibody to ALG-2 interacting protein X, suggests that for the microfilariae stage of *B*rugia *malayi* (which has no functional gut), EV are released from the excretory pore (Harischandra *et al*., 2018).

Uptake of parasitic nematode EV by mammalian cells was demonstrated for *H. polygyrus* (Buck *et al*., 2014) and for *B. malayi* (Zamanian *et al*., 2015) using labelled EV. Importantly, following exposure to *H. polygyrus* EV, alteration of gene expression in recipient cells was observed: treated cells showed down-regulation of immune-associated genes *il-33r* and *Dusp1*. In addition, specific miRNAs enriched within the *H. polygyrus* EV suppressed expression of a *Dusp*-3′-UTR reporter construct. This suggested, for the first time, that secreted parasitic nematode miRNAs may regulate host immune outcome. Studies using EV from *H. polygyrus* or *Trichuris muris* have also demonstrated the protective potential of parasitic nematode EV (Coakley *et al*., 2017; Shears *et al*., 2018). As EV may deliver small RNAs and proteins with immunoregulatory effects, vaccination has the potential to neutralize their functions and enhance immunity. Indeed, a reduction in worm burden of around 50% was observed following immunization of mice with EV purified from ES products of adult *H. polygyrus* or *T. muris*. Importantly, EV vaccination induced high levels of antibodies to EV and to ES supernatant, suggesting recognition of shared or cross-reactive epitopes that may limit infection. In addition, Coakley *et al*. (2017) detailed the suppressive effects of *H. polygyrus* EV on activation of both classical and alternatively stimulated macrophages. Importantly, they showed that exposure of macrophages to anti-EV antibodies abrogated this EV-mediated suppression. By tracking labelled EV within macrophage cells it was observed that, in the presence of anti-EV antibodies, EV localization was altered and led to accumulation within lysosomes, which reduced EV immunosuppressive effects. Whether the protective effect of EV vaccination may be mediated by neutralization of EV small RNA or protein function, or both, is currently unknown, however these studies are important in stimulating further investigation of the potential of EV to deliver parasite antigens for enhanced protection (Shears *et al*., 2018).

miRNAs released from GIN have not been identified in the serum or plasma of infected hosts (Buck *et al*., 2014; Britton *et al*., 2015), suggesting that they may act locally within the gastrointestinal tract. Consistent with this hypothesis, we were able to detect parasite-specific miRNAs in abomasal tissue and draining lymph nodes collected from *H. contortus*-infected, but not uninfected, sheep (Gu *et al*., 2017). Holz and Streit (2017) also failed to detect small RNAs of the GIN *Strongyloides ratti* in infected rat blood following infection. In contrast, parasite-derived miRNAs have been detected in the serum or plasma of hosts infected with tissue-dwelling parasitic nematodes, including the filariae *Dirofilaria immitis* (Tritten *et al*., 2014*a*), *Litomosoides sigmodontis* (Buck *et al*., 2014), *Onchocerca volvulus* and *Onchocerca ochengi* (Tritten *et al*., 2014*a*, 2104*b*; Quintana *et al*., 2015) or with the flatworm *Schistosoma japonicum* (He *et al*., 2013; Hoy *et al*., 2014). It was proposed that these released miRNAs may be involved in host–parasite interactions, although whether circulating parasite miRNAs have any effects on host gene expression are not yet known. Their release into the circulation has also stimulated interest in exploiting filarial and schistosome miRNAs as novel biomarkers of infection (reviewed in Cai *et al*., 2016 and Quintana *et al*., 2017). Parasite infection has also been shown to modulate expression of host miRNAs and many of these regulate genes involved in host innate and adaptive immune mechanisms. This has been detailed in previous reviews (e.g. Arora *et al*., 2017; Entwistle and Wilson, 2017). It is interesting to speculate on whether host miRNAs or other small RNAs could have any effect on the parasite. Uptake of labelled small RNAs by parasitic helminths maintained *in vitro* has been shown (e.g. Winter *et al*., 2014; Britton *et al*., 2016; Anandanarayanan *et al*., 2017). Whether sufficient levels of small RNAs could be transferred from the host to the parasite and whether these may be complexed to Ago or other RNA-binding proteins warrants further investigation to determine if host miRNAs could influence, for example, worm growth or reproduction.

Interestingly, recent work has shown that the widely-used anthelmintic drug ivermectin can reduce the release of EV from all life cycle stages of *B. malayi* (Mf, L3 and adult males and females) 24 h after exposure *in vitro* (Harischandra *et al*., 2018). The same approach showed a reduction in EV release from L3 of the related canine filarial nematode *D. immitis*, but not from an ivermectin resistant isolate, suggesting that the effect may be drug-specific (Harischandra *et al*., 2018). Ivermectin was previously shown to inhibit protein secretion by *B. malayi* microfilariae, leading to the hypothesis that one effect of ivermectin may be to reduce the release of immunosuppressive molecules from the excretory pore and thus enhance parasite clearance (Moreno *et al*., 2010). It is also possible that blocking the release and function of protein and/or small RNAs within EV may be responsible for this effect.

## Piwi-interacting RNAs

### Identification of piRNAs

While miRNAs repress mRNA translation by interacting with the Ago subfamily of Argonaute proteins, a different class of small RNA interacts with Piwi Argonautes (Seto *et al*., 2007). These are referred to as Piwi-interacting RNAs (piRNAs) of approximately 21–30 nucleotides in length that function mainly to silence mobile genetic elements, such as transposons. piRNAs have been identified from *C. elegans* and other animals, including *Drosophila* and mouse, and are required for fertility by protecting the germline from transposon insertion (Siomi *et al*., 2011).

The discovery that Piwi proteins interacted with small RNAs that were distinct from miRNA and siRNAs was first made in *Drosophila*. Sequencing of these piRNAs revealed that they mapped to retrotransposons and other repetitive sequences and were referred to as repeat-associated siRNAs (Saito *et al*., 2006). Parallel studies in mammals also identified Piwi-interacting small RNAs showing great complexity of sequence but with less identity to repetitive elements compared to those from flies (Lau *et al*., 2006). The diversity of piRNA sequences, both within and between species, makes their identification difficult, although in most species in which they have been identified, piRNAs are found clustered in the genome and are characterized by the presence of uracil as the 5′ nucleotide, with 5′ monophosphate and 2′-O-methyl 3′ termini (Brennecke *et al*., 2007).

While the function of piRNAs is conserved in different organisms, their biogenesis and mechanisms of silencing have diverged through evolution. In mouse and *Drosophila*, piRNAs are produced from long single-stranded precursors that are processed into multiple piRNA sequences (Brennecke *et al*., 2007). In contrast to miRNAs and siRNAs, processing does not depend in Dicer ribonuclease. Following the generation of initial primary piRNAs, sequences targeting active transposons are amplified by slicing of the target RNA to give rise to secondary piRNAs (referred to as the ping-pong cycle) (Gunawardane *et al*., 2007). It is thought that this amplification fine-tunes the piRNA response against active transposons (reviewed in Siomi *et al*., 2011).

### Nematode piRNAs

*Caenorhabditis elegans* piRNAs differ in several aspects to those in other animals. In *C. elegans*, mature piRNAs are characterized as 21 nucleotides in length that are produced from short precursors of 26–30 nucleotides expressed from individual genetic loci (Weick *et al*., 2014). They have a bias for uracil as the 5′ nucleotide (referred to as 21U-RNAs), in common with mature piRNAs in other species, and possess an upstream promoter motif (GTTTC) (Batista *et al*., 2008). Rather than slicing their target RNA sequence, *C. elegans* piRNAs direct binding of the piRISC to the target sequence, which initiates the synthesis of siRNA molecules complementary to the target (see Fig. 1). These siRNA molecules, referred to as 22G-RNAs, also mediate target gene silencing in the RNAi pathway (see below). Importantly, piRNAs are present in parasitic nematodes but only those of clade V. Their absence from other nematode clades stimulated a study to discover alternative pathways involved in transposon silencing (Sarkies *et al*., 2015). This identified 22G-RNAs, mapping antisense to transposons, in nematodes of clades III and IV, while a different mechanism, involving Dicer-dependent RNA-directed DNA methylation, functions in nematode clades I and II. Currently, it is not known why Piwi and piRNAs are maintained in clade V species.

The above features of *C. elegans* piRNAs are conserved in the clade V parasitic nematodes in which they have been identified, including *Pristionchus pacificus*, *H. contortus*, *H. polygyrus* and *Nippostrongylus brasiliensis*, based on small RNA sequencing and genome data (de Wit *et al*., 2009; Winter *et al*., 2012; Buck *et al*., 2014; Sarkies *et al*., 2015), although their organization is different. Two large clusters of piRNAs are found in *C. elegans* on chromosome IV (Ruby *et al*., 2006), while clustering of piRNA loci is not observed in *P. pacificus* (de Wit *et al*., 2009) nor in *H. contortus* (Winter *et al*., 2012). Recent work by Beltran *et al*. (2019) also identified differences in the chromatin structure of domains encoding piRNAs between nematode species, suggesting different modes of regulation. Two types of genomic organization of piRNA genes were characterized: P-type (e.g. *P. pacificus*) in which piRNA loci are found within active chromatin (H3K36me3), and C-type (e.g. *C. elegans*) where piRNA genes are in regions of repressive chromatin, associated with H3K27me3. How and why these different control mechanisms have evolved and whether they can co-exist are areas of ongoing investigation.

From *H. contortus* small RNA libraries, we identified >1000 reads representing putative piRNA sequences in adult worms, but not in L3 stage, consistent with a role in germline development and maintenance (Winter *et al*., 2012). piRNAs were not identified in *Brugia* (Winter *et al*., 2012) nor *A. suum* (Wang *et al*., 2011). We, and others, speculated that piRNAs may be required for adaptation/survival of progeny in varying environmental conditions, such as differences in temperature (Wang *et al*., 2011; Winter *et al*., 2012). However, piRNAs are not found in *Strongyloides* nematodes (clade IV) that have environmental larval stages (Holz and Streit, 2017). Sarkies *et al*. (2015) suggested that there may be clade V-specific transposable elements that require piRNA for silencing, rather than other small RNA pathways. This hypothesis is consistent with the differences in transposable element loads across nematode clades identified by Szitenberg *et al*. (2016). *Caenorhabditis elegans* mutants of the piRNA Argonaute PRG-1 show fertility defects and become sterile over many generations (Simon *et al*., 2014), demonstrating the importance of piRNAs in protecting the germline against transposon-mediated mutations. Interestingly, in gonochorist species, that mate every generation, there are greater numbers of piRNAs than in androdioecious species (self-fertilize) (Shi *et al*., 2013). This suggests that additional piRNAs may be required to defend against paternal transposons. Of potential relevance to this, female worms of the clade V parasitic nematodes *H. contortus and T. circumcincta* are known to be polyandrous (mate with multiple males) (Redman *et al*., 2008; Doyle *et al*., 2017). It is interesting to speculate that the piRNA pathway may be required in these clade V parasites to maintain genome viability in the face of high levels of paternal transposon mixing.

### Other functions of piRNAs

Recent small RNA sequencing has also identified differences in the number of piRNAs in different strains of *H. contortus* (Laing and Sarkies, unpublished data). Interestingly, this analysis compared *H. contortus* adult worms that are susceptible to anthelmintic drugs (MHco3.ISE) with a drug-resistant strain (MHco18.UGA). Whether these differences in piRNA levels have any consequence on drug sensitivity or may reflect different traits between the strains is not yet known.

While Piwi proteins and piRNAs localize predominantly to the germline, they have also been found in somatic stem cells. As germ cells and stem cells have the ability to replicate, the function of piRNAs in both may be to maintain genome integrity. The planarian flatworm *Schmidtea mediterranea* is used as model for stem cell proliferation and differentiation due to the presence of somatic stem cells (neoblasts) that allow tissue regeneration (Reddien and Sanchez Alvarado, 2004). Importantly, inhibition of *S. mediterranea* Piwi-encoding genes *smedwi-2* or *-3* resulted in lower abundance of piRNAs and failure of neoblast renewal and differentiation (Reddien *et al*., 2005). Studies in other flatworms and in cnidarians reported similar findings (De Mulder *et al*., 2009; Juliano *et al*., 2013). Piwi and piRNAs have been identified in lineage-restricted stem cells that do not give rise to germline cells, suggesting that they function to regulate gene expression and protect the genome in other cell types outside of the germline (reviewed in van Wolfswinkel, 2014). The progress being made in understanding the mechanisms and functions of piRNAs and Piwi proteins will help reveal new mechanisms of gene control.

## Small interfering RNAs (siRNAs)

siRNAs are approximately 21–25 bases in length and, like miRNAs, are derived from longer double-stranded RNA (dsRNA) molecules processed by Dicer. The dsRNA precursor can be derived from infective pathogens, such as viruses, endogenous genes, or be introduced artificially into cells to induce RNAi-mediated gene silencing. siRNAs bind to siRISC, with the antisense strand guiding the active RISC to its target mRNA, which is cleaved by Argonaute protein within RISC (Hammond *et al*., 2000) (Fig. 1). In contrast to miRNAs, which can potentially interact with hundreds of target mRNAs through seed sequence interaction, siRNAs bind with sequence complementarity along their entire length to direct specific gene silencing. The RNAi pathway was first identified in *C. elegans* (Fire *et al*., 1998) and shown to be a natural defense against viral infection (Felix *et al*., 2011). The discovery that siRNAs could also silence genes in mammalian cells (Elbashir *et al*., 2001) (but not dsRNA >30 bp, which induces an interferon response) stimulated great interest in developing these small RNAs as potential therapeutics. A number of siRNAs targeting specific genes of viruses or cancer have been tested (reviewed in Haussecker, 2012 and Chakraborty *et al*., 2017), although ensuring specificity of silencing, with no off target effects, and delivery of siRNAs into cells, can be challenging.

### RNAi in parasitic nematodes

Following the success of RNAi in *C. elegans*, a number of studies tested whether this approach could be applied as a functional genomics tool to identify novel drug and/or vaccine candidates for parasitic helminths (e.g. Hussein *et al*., 2002; Aboobaker and Blaxter, 2003; Skelly *et al*., 2003; Lustigman *et al*., 2004; Geldhof *et al*., 2006). While some parasite genes could be silenced, RNAi was not as effective as in *C. elegans*, particularly when dsRNA or siRNA was delivered by soaking (referred to as environmental RNAi). Our work in *H. contortus* showed that while some genes could be silenced using the soaking method, this was most effective for genes expressed in sites accessible to the environment, such as the gut, amphids and excretory cell. This suggested limited uptake and/or spreading of dsRNA in the non-feeding infective L3 stage used (Samarasinghe *et al*., 2011). This was supported by comparative genomic studies showing that while most genes required for siRNA-mediated gene silencing can be identified in parasitic nematodes (Dalzell *et al*., 2011; reviewed in Britton *et al*., 2016), a homologue of the transmembrane transporter SID-2, required for environmental RNAi in *C. elegans* (Winston *et al*., 2007), was absent in parasitic species.

More recent work has focused on delivering siRNA to the pre-infective L2 stage of *H. contortus*, which feed constitutively, in contrast to the more easily available, but non-feeding, infective L3 stage used previously. These recent studies by Blanchard *et al*. (2018) and Menez *et al*. (2019) were successful in determining a role for acetylcholine receptor subunit Hco-ACR-8 in mediating levamisole sensitivity and for Hco-NHR-8 in conferring tolerance to ivermectin, respectively. Their approach suggests that with improvements to the RNAi toolbox, including optimized delivery of siRNA, parasite stage examined, and phenotypic assays available, RNAi still holds promise for determining gene function. In addition, delivery by viral vectors may overcome the transient effect of exogenous siRNA or dsRNA. Effective gene silencing has been demonstrated following transfection of schistosome parasites with viral vectors expressing siRNAs (Hagen *et al*., 2014) and is being tested for the mouse GIN *N. brasiliensis* (Hagen and Selkirk, personal communication). Direct microinjection of siRNA or dsRNA can also be effective at gene silencing. The addition of lipofectamine to the microinjection mix used to deliver dsRNA was shown to enhance RNAi efficacy in a newly described nematode genus, *Auanema*, and to facilitate RNAi in an otherwise ‘resistant’ species, *P. pacificus* (Adams *et al*., 2019). These recent successes in determining function from RNAi phenotype, and development of new siRNA delivery methods, should stimulate further studies to progress from genome to function, in parallel with development of CRISPR/Cas9 gene editing for nematode species (Gang et al., 2017).

### Forms and functions of nematode siRNAs

From sequencing studies in *C. elegans*, different types of endogenous siRNAs have been identified. These are: 26G siRNAs, 26 nucleotides long with a bias for guanosine monophosphate at the 5′ end, produced by RNA-dependent RNA-polymerase (RdRP) RRF-3; and 22G siRNAs, 22 nucleotides long with a 5′ bias for guanosine triphosphate, produced by RdRPs RRF-1 and EGO-1 (reviewed in Almeida *et al*., 2019). 22Gs have been identified as the secondary siRNAs that are produced downstream of exogenous dsRNA, 26G RNAs and piRNAs (Fig. 1). They map antisense to target transcripts and, in *C. elegans*, are responsible for amplification of the RNAi response (Sijen *et al*., 2001). Interestingly, while siRNAs can silence target gene sequences within nematodes, recent work from the Buck lab suggests that siRNAs may also be involved at the host–parasite interface. Much of the data on small RNAs present within EVs have focused on miRNAs and their potential targets within host cells. However, Chow *et al*. (2019) generated small RNA datasets from EVs that included only 5′ monophosphate RNAs (miRNAs, piRNAs, 26G siRNAs) or alternatively, all small RNAs. The latter included those with 5′ triphosphate, using 5′ polyphosphatase treatment, not carried out in previous studies of EV small RNAs. This identified enrichment of 23G triphosphate secondary siRNAs (equivalent to *C. elegans* 22G siRNAs) in EVs from adult *Heligmosomoides bakeri* (*H. polygyrus*) and these were bound to the worm-specific Argonaute WAGO. Interestingly, these siRNAs mapped to recently evolved regions and repeat sequences in the parasite genome. It is not yet known if or how these may be taken up by host cells and what roles they may play either within the host or within the parasite.

## Conclusions and future directions

Genome data have enabled the identification of small RNA classes in parasitic nematodes. The challenge is to better understand the functions of these, both within the parasite and in host–parasite interactions.

With more advanced genome assembly and annotation, target identification based on miRNA–3′-UTR interaction, will improve. In addition, it is now feasible to sequence the transcripts expressed by each individual cell of an organism, including nematodes, using single cell RNA sequencing (Cao *et al*., 2017). Profiling of mRNAs and miRNAs in each cell can determine when and where these are expressed and, through inverse correlation analysis, identify potential regulatory functions (Wang *et al*., 2019). Microscopic or laser dissection of specific nematode tissues or cells, followed by mRNA and miRNA sequencing, is an alternative approach to reveal potential miRNA–mRNA networks. Dissection is possible with larger adult stage parasites and miRNA microarray profiling was successful for *H. contortus* gut tissue, isolated from adult female worms (Marks *et al*., 2019). However, confirmation of miRNA–mRNA interaction requires experimental verification, such as IP studies using antibodies to Argonaute proteins to isolate interacting complexes. While this has been achieved in *C. elegans*, only a few antibodies specific to parasite RNA binding proteins have been generated. These are available for ALG-2 interacting protein X (Harischandra *et al*., 2018) and extracellular WAGO, associated with EV of *H. bakeri* (Chow *et al*., 2019), but not yet for the major RISC complex Argonautes. IP studies, together with gene knockout approaches, would greatly advance knowledge of the specific interactions and functions of parasite small RNAs.

Little is currently known about how expression of small RNAs is regulated. Detailed studies in *C. elegans* and related species are beginning to reveal the mechanisms involved. Intergenic miRNAs have promoters similar to those of protein-coding genes and recent work has identified specific sequence motifs that determine expression pattern (Jovelin *et al*., 2016). As gene annotation improves, the same approach, using motif discovery tool MEME, can be applied to parasitic species. Interestingly, while miRNAs may be regulated by TFs, a number of miRNAs are known to target TFs, forming regulatory loops (Shalgi *et al*., 2007). Further details of the regulatory mechanisms and networks of miRNAs will help identify their control, function and evolution.

piRNAs target transposons and give rise to the 22G class of small RNAs. While 22G-RNAs are present in all nematode clades, piRNAs are restricted to clade V. It would be of interest to determine if this reflects a biological/genetic feature of this clade, such as mating behaviour, larger number of progeny, larger genome size for some species, and whether piRNAs are essential for fertility in parasitic species. It may be speculated that in sexually reproducing worms, and especially in polyandrous species, piRNAs may protect against male transposons to allow fertilization and development of progeny. Interestingly, Sargison *et al*. (2019) observed a reduced proportion of inter-strain hybrid F1 progeny developing following genetic mating of different *H. contortus* strains, relative to that observed following intra-strain mating. The underlying mechanisms of this post-zygotic incompatibility are as yet unknown but it would be important to determine if differences in expression of piRNAs or 22G-RNAs between strains could be responsible. As discussed above, approaches to enhance RNAi efficacy in parasitic nematodes are in progress; in the near future it may therefore be feasible to determine the roles of each class of small RNA, through knockout/knockdown of genes encoding Argonaute, RNA biogenesis or RNA binding proteins, such as PRG-1, required for piRNA function, as well as inhibition of specific small RNAs.

In addition to roles within parasitic nematodes, small RNAs, predominantly miRNAs, have been sequenced from EV released by parasites *in vitro* (Buck *et al*., 2014; Zamanian *et al*., 2015; Gu *et al*., 2017). As EV can be taken up by host cells, it is thought that these transported miRNAs play a role at the host–parasite interface. Development of tagged parasite miRNAs or knockout of miRNAs, combined with recipient host cell analysis, would be a useful approach to determine potential targets and functions within host cells. Studies to date have characterized EV released *in vitro*; whether EVs are released *in vivo* and transport the same RNAs and proteins is unknown. Evidence indicates release of miRNAs during infection with filarial (clade III) parasites and schistosomes, although it is not clear if these are released freely or may be derived from degraded EV or dying worms. Detailed analysis of small RNAs in EV of *H. bakeri* revealed, for the first time, the presence of 23G siRNAs (Chow *et al*., 2019). These are proposed to target repeat sequences and novel genomic regions of the parasite and were associated with Argonaute WAGO. While their abundance within EV may suggest host–parasite interaction, EV could also be a means of parasite–parasite communication. Determining the complete small RNA profile of EV from other parasitic nematode species, as well as *C. elegans*, and whether this changes following exposure to different environmental conditions may help determine if this is a novel and potentially important route of communication between worms.

In conclusion, recent studies have revealed novel mechanisms of small RNA regulation and packaging, helping advance our understanding of the diverse roles of these RNAs. Further efforts to effectively silence parasite genes by RNAi-mediated pathways will continue to reveal the importance of the different RNA classes in parasite development, communication, protection from invading genetic elements and in host–parasite interactions.
